# Performance of four HRP-2/pLDH combination rapid diagnostic tests and field microscopy as screening tests for malaria in pregnancy in Indonesia: a cross-sectional study

**DOI:** 10.1186/s12936-015-0943-5

**Published:** 2015-10-29

**Authors:** Rukhsana Ahmed, Elvira I. Levy, Sylvia S. Maratina, Judith J. de Jong, Puji B. S. Asih, Ismail E. Rozi, William Hawley, Din Syafruddin, Feiko ter Kuile

**Affiliations:** Department of Clinical Sciences, Liverpool School of Tropical Medicine, Pembroke Place, Liverpool, L3 5QA UK; Academic Medical Centre, University of Amsterdam, Amsterdam, The Netherlands; Malaria Laboratory, Eijkman Institute for Molecular Biology, Jakarta, Indonesia; UNICEF, Jakarta, Indonesia

**Keywords:** Malaria, Pregnancy, RDT, Histidine rich protein, Screening, Field microscopy, Indonesia

## Abstract

**Background:**

Malaria in pregnancy poses a major public health problem in Indonesia with an estimated six million pregnancies at risk of *Plasmodium falciparum* or *Plasmodium vivax* malaria annually. In 2010, Indonesia introduced a screen and treat policy for the control of malaria in pregnancy at first antenatal visit using microscopy or rapid diagnostic tests (RDTs). A diagnostic study was conducted in Sumba, Indonesia to compare the performance of four different RDTs in predominately asymptomatic pregnant women under field condition.

**Methods:**

Women were screened for malaria at antenatal visits using field microscopy and four HRP-2/pLDH combination RDTs (Carestart™, First-Response^®^, Parascreen^®^ and SD-Bioline^®^). The test results were compared with expert microscopy and nested PCR. End user experience of the RDTs in the field was assessed by questionnaire.

**Results:**

Overall 950 were recruited and 98.7 % were asymptomatic. The prevalence of malaria was 3.0–3.4 % by RDTs, and 3.6, 5.0 and 6.6 % by field microscopy, expert microscopy and PCR, respectively. The geometric-mean parasite density was low (*P. falciparum* = 418, *P. vivax* = 147 parasites/µL). Compared with PCR, the overall sensitivity of the RDTs and field microscopy to detect any species was 24.6–31.1 %; specificities were >98.4 %. Relative to PCR, First-Response^®^ had the best diagnostic accuracy (any species): sensitivity = 31.1 %, specificity = 98.9 % and diagnostic odds ratio = 39.0 (DOR). The DOR values for Carestart™, Parascreen^®^, SD-Bioline^®^, and field microscopy were 23.4, 23.7, 23.5 and 29.2, respectively. The sensitivity of Pan-pLDH bands to detect PCR confirmed *P. vivax* mono-infection were 8.6–13.0 %. The sensitivity of the HRP-2 band alone to detect PCR confirmed *P. falciparum* was 10.3–17.9 %. Pan-pLDH detected *P. falciparum* cases undetected by the HRP-2 band resulting in a better test performance when both bands were combined. First Response^®^ was preferred by end-users for the overall practicality.

**Conclusion:**

The diagnostic accuracy to detect malaria among mostly asymptomatic pregnant women and perceived ease of use was slightly better with First-Response^®^, but overall, differences between the four RDTs were small and performance comparable to field microscopy. Combination RDTs are a suitable alternative to field microscopy to screen for malaria in pregnancy in rural Indonesia. The clinical relevance of low density malaria infections detected by PCR, but undetected by RDTs or microscopy needs to be determined.

## Background

Malaria in pregnancy poses a major public health problem in Indonesia, where annually an estimated 6.3 million pregnancies are potentially at risk of *Plasmodium falciparum* or *Plasmodium vivax* malaria [1] and the corresponding risk of malaria associated maternal anaemia and low birth weight due to pre-term delivery or intra-uterine growth retardation [2–4]. Early detection and effective management of asymptomatic and symptomatic malaria is essential to reduce the burden of malaria in pregnancy.

Indonesia introduced a screen and treat policy for the control of malaria in pregnancy in 2010 [5]. It consists of screening all pregnant women for malaria at their first antenatal visit using microscopy or rapid diagnostic test (RDT), regardless of the presence or absence of symptoms. At subsequent antenatal visits, testing for malaria is done only in women with symptoms of malaria. Malaria positive women are treated with artemisinin-based combination therapy (ACT, dihydroartemisinin–piperaquine), except in the first trimester when quinine is used. Microscopy, remains the gold standard for malaria diagnosis in most health facilities. RDTs are used in the village based antenatal clinics.

The common immuno-chromatography based malaria RDTs detect the histidine rich protein-2 (HRP-2) antigen or parasite lactate dehydrogenase (pLDH) enzyme. The HRP-2/pLDH combination RDTs are commonly used in Indonesia and other Asian countries where multiple *Plasmodium* species are endemic. The HRP-2 antigen is specific for *P. falciparum* and Pan-pLDH detects all human infecting species [6, 7].

RDTs are essential for the success of the malaria in pregnancy screening programme. Numerous studies have shown the ability of HRP-2 based RDTs to detect *P. falciparum* in symptomatic population in areas of varying transmissions [8–13]. Few studies have evaluated RDTs in pregnancy [14–16] and fewer as a screening test in pregnant women [17–20], specially to detect *P. vivax* in asymptomatic women [21–23], and under field conditions. The study aimed to assess the ability of RDTs to detect *P. falciparum* and *P. vivax,* in mostly asymptomatic pregnant women and to compare it against the performance of standard field microscopy. For this, a cross-sectional study was conducted in eastern Indonesia comparing four combination RDTs and field microscopy against expert microscopy and PCR to identify the RDT with highest diagnostic accuracy and field practicality to use as a screening test in rural antenatal clinics.

## Methods

### Study area and facilities

The study was conducted between March and July 2012 in south-west Sumba district in eastern Indonesia where malaria transmission is low and seasonal and varies substantially across sub-districts [24]. Prevalence of malaria in the general population (all age groups) in the rainy season (December–March) is 6.8 % with a predominance of *P. falciparum*. In the dry season (April–November) both *P. falciparum* and *P. vivax* are present in equal proportions with an overall prevalence of 4.9 % by PCR.

The study was conducted in ‘*Posyandus*’, which are village based community integrated activities held monthly that include provision of outreach antenatal care services provided by the primary health centres (*Puskesmas*). A total of 45 *Posyandus* were involved in the study, served by four *Puskesmas* in the sub-districts of Bondo-Kodi, Kori, Wallandimu and Pannengo-ede, each covering a catchment population of approximately 30,000 people and 10–11 villages.

### Participant enrolment

Pregnant women of any gravidity aged between 15 and 49 years who attended the *Posyandu* for routine antenatal care were enrolled. A questionnaire consisting of socio-demographic information, obstetric history, history of fever and drug use, including malaria prevention measures was completed and the axillary temperature recorded. A finger prick blood sample was taken to simultaneously test the four RDTs, malaria microscopy and dried blood spots for PCR. Women testing positive for malaria with any RDT were treated according to the national policy which was a 3 day course of ACT (dihydroartemisinin–piperaquine) in the second and third trimester and 7 days of oral quinine in the first trimester.

### Microscopy

Thick and thin smears were made on the same slide and stained with 5 % Giemsa. For the ‘field microscopy’, the smears were read by the staff microscopist at each of the four *Puskesmas* who were unaware of the RDT results. Thin smears were used to identify species. Expert microscopy consisted of re-examination of all the slides by the senior microscopist at the Malaria Laboratory-1, Eijkman Institute in Jakarta, who was blinded to field microscopy and RDT results. In cases of disagreement between field and expert microscopy, PCR result was taken as final. A slide was declared negative if parasites were absent after examining 200 high power fields. Parasite density was quantified against 300 leukocytes on an assumed leukocyte count of 8000 per µL of blood. Sub-microscopic infection was defined as parasites detected by PCR, but not by expert microscopy or RDT respectively.

### Rapid diagnostic tests

The four RDTs included Parascreen Rapid Test Pan/Pf^®^ (Zephyr Biomedical System, India, Catalogue No: 50310025), SD Bioline Malaria Ag Pf/Pan^®^ (Standard Diagnostic Inc. Suwon City, South Korea, Catalogue No: 05FK60), the two RDTs used in the malaria control programme in Indonesia at the time of the study, and First Response Malaria pLDH/HRP-2 combo^®^ (Premier Medical Corporation Ltd, India, Catalogue No: l16FRC30) and CareStart Malaria pLDH/HRP2 combo™ (Access Bio Inc., NJ, USA, Catalogue No: G0131), two of the best performing RDTs indicated in the WHO/FIND round 1–3 report [25]. Trained study staff performed and interpreted the RDTs according to the manufacturer’s instructions.

Accordingly, four drops of buffer were used for SD Bioline^®^ and read within 15 min; for the other three RDTs two drops of buffer were used and was read within 20 min. Malaria positivity was defined if any of the HRP-2 or pLDH bands were visible (plus the control band). If only HRP-2 band plus control band was visible it was considered as a *P. falciparum* infection and visibility of only Pan-pLDH band was considered a *P. vivax* infection. When both bands were positive simultaneously it was considered a *P. falciparum* infection or a mixed infection.

### Polymerase chain reaction

Nested PCR was performed on all samples to detect malaria parasites and for speciation. A blood sample was spotted on Whatman grade-1 filter paper, air-dried, placed in a plastic bag and transported to Jakarta. DNA was extracted with a 20 % Chelex solution and stored at −20 °C. Nested PCR based on the principle described by Snounou et al. [26] was used for genus and species specific analysis of *P. falciparum* and *P. vivax*. All ribosomal PCR positives samples were confirmed using mitochondrial DNA based PCR. Repeat mitochondrial PCR was performed for discordant *P. vivax* samples using KAPA2G™ Fast ReadyMix (2×) (KAPA Bio systems) containing all components for the PCR and the primers MitoPf-F 307 and MitoPf-R 5904 and adding 2 µL of DNA template. Species identification was done on PCR-Restriction Fragment Length Polymorphism (*Rflp*) using the restriction endonuclease enzyme *Acl*1. The amplicon was electrophoresed on 2 % agarose gel and the species specific DNA bands were viewed. Staff unaware of the microscopy and the RDT results performed PCR.

### Quality control measures and RDT transport and storage

Room temperature and humidity in the RDT storage rooms in the *Puskesmas* were monitored and recorded using Tinytag™ Data Loggers with alarms set at 40 °C, the upper limit of temperature stability for SD Bioline^®^. The recorded data was saved into the main database and verified fortnightly. The maximum humidity recorded was 82.1 rh % and temperature ranged between 22.8 and 28.5 °C during the study period. To follow real-life field situations, environmental conditions in storage facilities were not controlled although excessive exposure to direct sunlight was avoided during RDT transportation. Individual kits were opened only at the time of testing and were checked for the presence of the desiccant. A single batch of each RDT with same LOT number purchased directly from the manufacturer was used.

### End user experience

The ease of use and practicality of the study RDTs in the field situation was explored mid-way of the study. The *Puskesmas* midwives/nurses trained for the study were given a questionnaire to assess the readability and print clarity of information on the package exterior, ease of using the blood collecting devices, transferring blood to the RDT cassette and addition of buffer drops. Each parameter was scaled from easy, moderately easy and hard and the response frequency to each category calculated.

### Sample size

A sample size of 940 pregnant women were estimated to be sufficient to compare RDTs with expert microscopy and PCR. Allowing for a 10 % loss of samples or unsuccessful tests a target sample size of 1045 women was estimated. An anticipated malaria prevalence of 10 % detectable by PCR was considered with reference to a previous study in the area [27]. This assumption would allow the detection of a sensitivity and specificity of 0.97 with a lower 95 % confidence limit >0.9 with 95 % probability.

### Statistical analysis

Data were double entered and verified using XAMPP Windows 1.7.7. Frequencies and proportions were calculated using SPSS version 20.0. The results of the four RDTs and field microscopy were first compared against expert microscopy as the reference test. In subsequent analysis the RDTs, field and expert microscopy were compared against PCR (all samples) as the reference test, for any species (overall) and by specific RDT bands (HRP-2 and pLDH) against the species identified by PCR. For both sets of analyses, the sensitivity, specificity, positive and negative predictive values and likelihood ratios (LR) were calculated with corresponding 95 % confidence intervals (CI) using an online calculator which uses the efficient score method to calculate 95 % CI suitable for situations where the proportion is small and prevalence is low [28]. The diagnostic odds ratio (DOR) was used as a single summary indicator of test effectiveness independent of prevalence combining sensitivity and specificity, where DOR was defined as the ratio of the odds of positivity in those with malaria (defined by PCR or expert microscopy) relative to the odds of positivity in those without malaria. The DOR is also the ratio of the positive and negative likelihood ratio (LR+/LR−) [29].

### Ethical approval

The study was approved by the Research and Ethical Committees of the Eijkman Institute for Molecular Biology, Indonesia and the Liverpool School of Tropical Medicine, UK. Written informed consent was obtained.

## Results

A total of 950 women who attended the study *Posyandus* for antenatal care were enrolled between March and July 2012. Most women were indigenous to southwest Sumba (97.5 %) and lived in rural areas (100 %). Their mean age (SD) was 28.8 (6.2) years and 17.8 % were primigravidae. The proportion of women reporting ownership of long lasting insecticide treated nets (LLINs) was 29.9 % of whom 93.3 % reported to have slept under the net the previous night. A documented fever (≥37.5 °C) was present in 12 of 949 women (1.3 %) and 157 (16.5 %) reported a history of fever in the previous week of whom 31 (19.7 %) reported taking anti-malarial drugs.

### Malaria prevalence detected by RDTs, microscopy and PCR

Overall a full set of results for the RDTs, blood smears and PCR samples were available from 934 of the 950 women. The remaining 16 women (1.7 %) were excluded from analysis because not all four RDT tests were successful or no microscopy results were available.

Malaria positivity by RDT (HRP2 or pLDH) ranged from 3.0 % (28 women) with Carestart™ to 3.4 % (32 women) with Parascreen^®^ (Table 1). The higher number of positives with Parascreen^®^ relative to Carestart™ was due to a higher number of positives by the HRP-2 band (21 versus 15). Overall, differences in results were greatest for the single Pan-pLDH band (which in the absence of a positive HRP-2 band is indicative of non-falciparum malaria infections) and positivity varied from 6.6 % with SD Bioline^®^ to 21.4 % with Carestart™.

**Table 1:** Malaria detected by the four RDTs (HRP-2 and pLDH bands), microscopy and PCR

	Carestart™	First Response^®^	Parascreen^®^	SD Bioline^®^
	n = 934	n = 934	n = 934	n = 934
RDT positive (any band) n (%)	28 (3.0)	30 (3.2)	32 (3.4)	30 (3.2)
HRP-2 band only, n (%)	15 (53.6)	17 (56.6)	21 (65.6)	20 (66.6)
*P. falciparum* *confirmed by PCR*				
HRP-2 band, n (%)	4 (26.7)	7 (41.2)	7 (33.3)	6 (30.0)
Pan-pLDH band only, n (%)	6 (21.4)	6 (19.4)	4 (12.5)	2 (6.6)
*Species confirmed by PCR*				
* P. falciparum*, n (%)	2 (33.0)	3 (50.0)	1 (25.0)	1 (50.0)
* P. vivax*, n (%)	2 (33.0)	2 (33.0)	2 (50.0)	1 (50.0)
Negative	2 (33.0)	1 (16.9)	1 (25.0)	0
HRP2 + pLDH bands, n (%)	7 (25.0)	7 (23.0)	7 (21.8)	8 (26.6)
*Species confirmed by PCR*				
* P. falciparum,* n (%)	5 (71.4)	5 (71.4)	5 (71.4)	6 (75.0)
* P. vivax,* n (%)	1 (14.3)	1 (14.3)	1 (14.3)	1 (12.5)
Mixed (*Pf *+ *Pv*), n (%)	1 (14.3)	1 (14.3)	1 (14.3)	1 (12.5)

	Field microscopy	Expert microscopy	PCR n = 934	
	n = 934	n = 934	Overall	Sub-microscopic^a^
Parasitaemia any, n (%)	34 (3.6)	47 (5.0)	62 (6.6)	32 (3.4)
*P. falciparum*, n (%)	26 (76.5)	24 (51.1)	32 (51.6)	20 (62.5)
*P. vivax*, n (%)	8 (23.5)	13 (27.7)	23 (37.0)	12 (37.5)
*P. malariae*, n (%)	–	5 (10.6)	–	–
Mixed (*Pf* + *Pv*) n (%)	–	5 (10.6)	7 (11.3)	–

*Pf*, *P. falciparum*; *Pv*, *P. vivax*

^a^Sub-microscopic = expert microscopy negative, PCR positive

There were 34 (3.6 %) and 47 (5.0 %) malaria (any species) positives detected by field and expert microscopy respectively when both asexual and sexual stages were considered. Overall, 5 of the 34 (14.7 %) and 8 of the 47 (17.0 %) were positive for gametocytes by field and expert microscopy respectively. Expert microscopy detected 5 (10.6 %) mono infections with *Plasmodium malariae* and 5 (10.6 %) mixed infections with *P. falciparum* and *P. vivax*.

Overall infection prevalence by PCR was 6.6 % (all species) and 3.9 % were sub-patent infections (microscopy or RDT negative). Of the 62 positives identified by PCR, 39 (62.9 %) were with *P. falciparum* (32 mono-infections and seven mixed with *P. vivax*), and 23 (37.0 %) were *P. vivax* mono-infections. No infections with *P. malariae* were detected.

There were 31 women who had reported to have taken antimalarial drug in the previous week. Out of these, one women had all four RDTs positives, two women had a positive microscopy (6.4 %) and three women had a positive PCR (9.6 %) for any malaria.

### Discriminating ability of the RDTs and field microscopy

#### PCR as reference test

*Malaria overall* (*any species*) (Table 2): The sensitivity and specificity of the RDTs to detect malaria (any species) (positive HRP-2 or Pan-pLDH band) against PCR was relatively similar for all four RDTs. The sensitivity ranged between 25.8 and 32.3 %. The specificity was >98.3 % for all RDTs. First Response^®^ had the highest combination of sensitivity and specificity, reflected in the highest LR-positive, lowest LR-negative scores and the highest DOR value (41.0, 95 % CI 16.9–101.2). The DOR of Carestart™ (24.9), Parascreen^®^ (−25.0) and SD Bioline^®^ (24.9) were similar. The sensitivity of field microscopy was comparable to First Response^®^, but the specificity and the DOR (29.1) was lower.

**Table 2:** Accuracy indices of the RDTs and Field microscopy compared with expert microscopy and PCR to detect any malaria (HRP-2 or pLDH band; *P. falciparum* or *P. vivax*)

	Carestart™	First Response^®^	Parascreen^®^	SD Bioline^®^	Field microscopy	Expert microscopy
*Expert microscopy as reference test*						
TP, FP	20, 8	21, 9	21, 11	22, 8	21, 13	N/A
FN, TN	27, 879	26, 878	26, 876	25, 879	26, 874	
Sensitivity (95 % CI)	42.6 (31.5–50.9)	44.7 (33.4–53.5)	44.6 (30.4–59.7)	46.8 (35.6–55.1)	44.7 (32.9–54.9)	
Specificity (95 % CI)	99.1 (98.5–99.5)	99.0 (98.4–99.5)	98.7 (97.7–99.3)	99.1 (98.5–99.5)	98.5 (97.9–99.1)	
PPV (95 % CI)	71.4 (52.9–85.4)	70.0 (52.3–83.8)	65.6 (46.7–80.8)	73.3 (55.8–86.3)	61.8 (45.5–75.8)	
NPV (95 % CI)	97.0 (96.4–97.5)	97.1 (96.5–97.6)	97.1 (95.7–98.0)	97.2 (96.7–86.3)	97.1 (96.5–97.6)	
LR positive (95 % CI)	47.2 (21.2–110.3)	44.0 (20.7–97.3)	36.0 (18.4–70.3)	51.9 (23.8–119.4)	30.4 (15.7–59.3)	
LR negative (95 % CI)	0.58 (0.49–0.69)	0.56 (0.47–0.67)	0.56 (0.43–0.72)	0.54 (0.45–0.65)	0.56 (0.46–0.68)	
DOR (95 % CI)	81.4 (30.5–223.4)	78.7 (30.6–207.9)	64.3 (26.3–168.8)	96.7 (36.4–264.7)	54.3 (27.9–130.1)	
*PCR as the reference test*						
TP, FP	16, 12	20, 10	18, 14	17, 13	20, 14	30, 17
FN, TN	46, 860	42, 862	44, 858	45, 859	42, 858	32, 855
Sensitivity (95 % CI)	25.8 (15.8–38.7)	32.3 (21.2–45.4)	29.0 (18.5–42.1)	27.4 (17.2–40.4)	32.2 (17.2–40.4)	48.4 (35.6–61.3)
Specificity (95 % CI)	98.6 (97.5–99.2)	98.8 (97.8–99.4)	98.3 (97.2–99.0)	98.5 (97.3–99.1)	98.3 (97.2–99.0)	98.0 (96.8–98.8)
PPV (95 % CI)	57.1 (37.4–74.9)	66.6 (47.1–82.0)	56.2 (37.8-73.1)	56.6 (37.6–74.0)	58.8 (40.8–74.8)	63.8 (48.4–76.9)
NPV (95 % CI)	94.9 (93.2–96.2)	95.3 (93.7–96.5)	95.1 (93.4–96.3)	95.0 (93.3–96.3)	95.3 (93.6–96.5)	96.3 (94.8–97.4)
LR positive (95 % CI)	18.7 (9.2–37.8)	28.2 (13.7–57.4)	18.8 (9.4–34.6)	18.3 (9.3–36.1)	20.0 (10.6–37.8)	24.8 (14.5–42.4)
LR negative (95 % CI)	0.75 (0.65–0.87)	0.68 (0.57–0.81)	0.72 (0.61–0.84)	0.73 (0.63–0.85)	0.68 (0.57–0.81)	0.52 (0.41–0.69)
DOR (95 % CI)	24.9 (10.4–60.1)	41.0 (16.9–101.2)	25.0 (11.0–57.4)	24.9 (10.7–58.6)	29.1 (12.9–66.1)	47.1 (22.4–100.2)

*TP* true positive, *FP* false positive, *FN* false negative, *TN* true negative, *PPV* positive predictive value, *NPV* negative predictive value, *LR* likelihood ratio, *DOR* diagnostic odds ratio

*Plasmodium falciparum and single HRP*-*2 band* (Table 3): The performance of the HRP-2-bands alone (i.e. ignoring the results of the Pan-pLDH band) to detect *P. falciparum* infections assessed by PCR, showed that the overall sensitivity ranged from 10.3 % with Carestart™ to 17.9 % with Parascreen^®^ and First Response^®^. The PPV and DOR indices were highest for First Response^®^ reflected by less false positives compared to other RDTS.

**Table 3:** Accuracy indices of the HRP-2 band and Field microscopy compared with expert microscopy and PCR to detect *P. falciparum*

	Carestart™	First Response^®^	Parascreen^®^	SD Bioline^®^	Field microscopy	Expert microscopy
*Expert microscopy as reference test*						
TP, FP	7, 8	9, 8	8, 13	9, 11	13, 13	N/A
FN, TN	17, 902	15, 902	16, 897	15, 899	11, 897	
Sensitivity (95 % CI)	29.2 (13.4–51.2)	37.5 (19.6–59.2)	33.3 (16.4–55.3)	37.5 (19.6–59.2)	54.2 (33.2–73.8)	
Specificity (95 % CI)	99.1 (98.2–99.6)	99.1 (98.2–99.6)	98.6 (97.5–99.2)	98.8 (97.8–99.4)	98.6 (97.5–99.2)	
PPV (95 % CI)	46.7 (22.3–72.6)	52.9 (28.5–76.1)	38.1 (19.0–61.3)	45.0 (23.8–67.7)	50.0 (30.4–69.6)	
NPV (95 % CI)	98.2 (97.0–98.9)	98.4 (97.3–99.0)	98.2 (97.1–99.0)	98.4 (97.2–99.0)	98.8 (97.8–99.4)	
LR positive (95 % CI)	33.2 (13.1–84.1)	42.7 (18.0–101.0)	23.3 (10.7–51.0)	31.0 (14.2–67.8)	37.9 (19.7–72.9)	
LR negative (95 % CI)	0.71 (0.55–0.92)	0.63 (0.46–0.86)	0.68 (0.51–0.90)	0.63 (0.46–0.86)	0.46 (0.30–0.72)	
DOR (95 % CI)	46.4 (13.3–163.3)	67.7 (20.4–228.7)	34.5 (11.2–105.7)	49.0 (15.9–152.9)	81.5 (30.0–243.0)	
*PCR as the reference test*						
TP, FP	4, 11	7, 10	7, 14	6, 14	12, 14	12, 12
FN, TN	35, 884	32, 885	32, 881	33, 881	27, 881	27, 883
Sensitivity (95 % CI)	10.3 (3.3–25.2)	17.9 (8.1–34.1)	17.9 (8.1–34.1)	15.4 (6.4–31.2)	30.8 (17.5–47.7)	30.8 (17.5–47.7)
Specificity (95 % CI)	98.8 (97.7–99.4)	98.9 (97.9–99.4)	98.4 (97.3–99.1)	98.4 (97.3–99.1)	98.4 (97.3–99.1)	98.7 (97.6–99.2)
PPV (95 % CI)	26.7 (8.9–55.2)	41.2 (19.4–66.5)	33.3 (15.5–56.9)	30.0 (12.8–54.3)	46.2 (27.1–66.3)	50.0 (29.6–70.4)
NPV (95 % CI)	96.2 (94.7–97.3)	96.5 (95.1–97.6)	96.5 (95.0–97.6)	96.4 (94.9–97.5)	97.0 (95.6–98.0)	97.0 (95.7–98.0)
LR positive (95 % CI)	8.3 (2.8–25.0)	16.1 (6.5–40.0)	11.5 (4.9–26.8)	9.8 (4.0–24.2)	19.7 (9.8–39.7)	22.9 (11.0–47.8)
LR negative (95 % CI)	0.9 (0.82–1.01)	0.83 (0.72–0.96)	0.83 (0.72–96.5)	0.86 (0.75–0.98)	0.70 (0.57–0.87)	0.70 (0.57–0.87)
DOR (95 % CI)	9.2 (2.3–33.4)	19.4 (6.2–59.9)	13.8 (4.7–39.8)	11.4 (3.7–34.6)	30.0 (10.9–71.9)	32.7 (12.4–86.9)

*TP* true positive, *FP* false positive, *FN* false negative, *TN* true negative, *CI* confidence interval, *PPV* positive predictive value, *NPV* negative predictive value, *LR* Likelihood ratio, *DOR* diagnostic odds ratio

*Plasmodium falciparum and single Pan*-*pLDH band* (Table 4): The accuracy of pLDH band alone to detect *P. falciparum* was assessed against PCR detected mono- *P. falciparum* (after exclusion of *P. vivax* mono or mixed infection from the analysis). The sensitivity ranged from 18.8 % with Parascreen^®^ to 25.0 % with First Response^®^. The DOR was highest for SD Bioline^®^ followed by First Response^®^.

**Table 4:** Accuracy indices of the pLDH band compared with expert microscopy and PCR to detect *P. falciparum*

	Carestart™	First Response^®^	Parascreen^®^	SD Bioline^®^
*Expert microscopy as the reference*				
TP, FP	7, 6	6, 7	6, 5	6, 4
FN, TN	17, 904	18, 903	18, 905	18, 906
Sensitivity (95 % CI)	29.2 (13.4–51.2)	25 (10.6–47.1)	25 (10.6–47.1)	25 (10.6–47.1)
Specificity (95 % CI)	99.3 (98.5–99.7)	99.2 (98.3–99.7)	99.5 (98.6–99.8)	99.6 (98.8–99.9)
PPV (95 % CI)	53.8 (26.1–79.6)	46.1 (20.4–73.9)	54.5 (24.6–81.9)	60 (27.4–86.3)
NPV (95 % CI)	98.2 (96.9–98.8)	98.0 (96.7–98.8)	98 (96.9–98.8)	98.1 (96.9–98.8)
LR positive (95 % CI)	44.2 (16.1–121.7)	32.5 (11.8–89.4)	45.5 (14.9–138.8)	56.9 (17.2–188.5)
LR negative (95 % CI)	0.71 (0.55–0.92)	0.76 (0.60–0.95)	0.75 (0.60–0.95)	0.75 (0.60–0.95)
DOR (95 % CI)	62 (16.5–239.0)	43 (11.4–162.7)	60.3 (14.5–257.2)	75.5 (16.9–356.3)
*PCR as the reference*				
TP, FP	7, 6	8, 5	6, 5	7, 3
FN, TN	25, 896	24, 897	26, 897	25, 899
Sensitivity (95 % CI)	21.9 (9.9–40.4)	25 (12.1–43.8)	18.8 (7.9–37.0)	21.9 (9.9–40.4)
Specificity (95 % CI)	99.3 (98.4–99.7)	99.4 (98.6–99.8)	99.4 (98.6–99.8)	99.7 (98.9–99.9)
PPV (95 % CI)	53.8 (26.1–79.6)	61.5 (32.2–84.9)	54.5 (24.6–81.9)	70 (35.4–91.9)
NPV (95 % CI)	97.3 (96.0–98.2)	97.4 (96.1–98.3)	97.2 (95.8–98.1)	97.3 (96.0–98.2)
LR positive (95 % CI)	32.9 (11.7–92.3)	45.1 (15.6–130.2)	33.8 (10.9–105.1)	65.8 (17.8–242.7)
LR negative (95 % CI)	0.79 (0.65–0.94)	0.75 (0.62–0.92)	0.82 (0.69–0.97)	0.78 (0.65–0.94)
DOR (95 % CI)	41.8 (11.5–154.1)	59.8 (16.2–230.7)	41.4 (10.3–169.9)	83.9 (18.0–439.7)

*TP* true positive, *FP* false positive, *FN* false negative, *TN* true negative, *CI* confidence interval, *PPV* positive predictive value, *NPV* negative predictive value, *LR* likelihood ratio, *DOR* diagnostic odds ratio

*Plasmodium falciparum and the combined HRP*-*2 and Pan*-*pLDHbands* (Table 5): The accuracy of HRP-2 and pLDH positive bands to detect *P. falciparum* was evaluated with PCR confirmed *P. falciparum* (any). The sensitivities ranged between 30.7 and 41.0 %. The LR positive and DOR values ranged between 17.2 and 24.5 % for Carestart™ and 26.2 and 44.4 % for First Response^®^, respectively.

**Table 5:** Accuracy indices of the RDTs (HRP-2 and pLDH) bands compared with PCR to detect *P. falciparum*

	Carestart	First Response	Parascreen	SD Bioline
*PCR as reference test*				
TP, FP	12, 16	16, 14	14, 18	14, 16
FN, TN	27, 879	23, 881	25, 887	25, 879
Sensitivity (95 % CI)	30.7 (17.5–47.7)	41.0 (25.9–57.8)	35.8 (21.6–52.8)	35.8 (21.6–52.8)
Specificity (95 % CI)	98.2 (97.0–98.9)	98.4 (97.3–99.1)	97.9 (96.7–98.7)	98.2 (97.0–98.9)
PPV (95 % CI)	42.8 (25.0–62.5)	53.3 (34.6–71.2)	43.7 (26.8–62.1)	46.6 (28.7–65.3)
NPV (95 % CI)	97.0 (95.6–97.9)	97.4 (96.1–98.3)	97.2 (95.8–98.1)	97.2 (95.8–98.1)
LR positive (95 % CI)	17.2 (8.7–33.8)	26.2 (13.8–49.8)	17.8 (9.6–33.1)	20.1 (10.6–38.1)
LR negative (95 % CI)	0.7 (0.44–1.28)	0.59 (0.46–0.78)	0.65 (0.52–0.83)	0.65 (0.52–0.82)
DOR (95 % CI)	24.5 (9.7–61.2)	44.4 (17.8–108.9)	27.3 (11.3–65.6)	30.9 (12.5–75.5)

*TP* true positive, *FP* false positive, *FN* false negative, *TN* true negative, *CI* confidence interval, *PPV* positive predictive value, *NPV* negative predictive value, *LR* likelihood ratio, *DOR* diagnostic odds ratio; include positives for mixed species

*Plasmodium vivax and Pan*-*pLDH band* (Table 6): The performance of RDT to detect *P. vivax* was evaluated with PCR detected *P. vivax* mono-infections (after exclusion of *P. falciparum* mono or mixed infection from the analysis). The sensitivity to detect *P. vivax* was lower than for *P. falciparum* ranging from 8.7 % with SD Bioline^®^ to 13.0 % with the other three RDTs. The DOR values ranged from 10.8 % with SD Bioline^®^ to 16.9 % with Parascreen^®^. The field microscopy indices were relatively better for *P. vivax* detection with higher DOR values.

**Table 6:** Accuracy indices of the pLDH bands and Field microscopy compared with expert microscopy and PCR for the detection of *P. vivax*

	Carestart™	First Response^®^	Parascreen^®^	SD Bioline^®^	Field microscopy	Expert microscopy
*Expert microscopy as reference test*						
TP, FP	3, 10	2, 11	2, 9	2, 8	0, 8	N/A
FN, TN	19, 911	11, 910	11, 912	11, 913	13, 913	
Sensitivity (95 % CI)	13.6 (3.6–36.0)	15.4 (2.7–46.3)	15.4 (2.7–46.3)	15.4 (2.7–46.3)	0	
Specificity (95 % CI)	98.9 (97.9–99.4)	98.8 (97.8–99.4)	99.0 (98.1–99.5)	99.1 (98.2–99.6)	99.1 (98.2–99.6)	
PPV (95 % CI)	23.1 (6.2–54.0)	15.4 (2.7–46.3)	18.2 (3.2–52.2)	20.0 (3.5–55.8)	0	
NPV (95 % CI)	98.0 (96.8–98.7)	98.8 (97.8–99.4)	98.8 (97.8–99.4)	98.8 (97.8–99.4)	98.6 (97.5–99.2)	
LR positive (95 % CI)	12.6 (6.6–68.3)	12.9 (3.2–52.4)	15.7 (3.8–65.9)	17.7 (4.2–75.5)	0	
LR negative (95 % CI)	0.87 (0.74–1.03)	0.86 (0.68–1.08)	0.85 (0.68–1.08)	0.85 (0.67–1.07)	1 (1.0–1.0)	
DOR (95 % CI)	14.4 (2.9–63.7)	15.0 (2.0–86.3)	18.4 (2.4–109.8)	20.8 (2.7–126.8)	0	
*PCR as the reference test*						
TP, FP	3, 10	3, 10	3, 8	2, 8	4, 4	11, 2
FN, TN	20, 901	20, 901	20, 903	21, 903	19, 907	12, 909
Sensitivity (95 % CI)	13.0 (3.4–34.7)	13.0 (3.4–34.7)	13.0 (3.4–34.7)	8.7 (1.5–29.5)	17.4 (5.7–39.5)	47.8 (27.4–68.9)
Specificity (95 % CI)	98.9 (97.9–99.4)	98.9 (97.9–99.4)	99.1 (98.2–99.6)	99.1 (98.2–99.6)	99.6 (98.8–99.9)	99.8 (99.1–100.0)
PPV (95 % CI)	23.1 (6.2–54.0)	23.1 (6.2–54.0)	27.3 (7.3–60.7)	20.0 (3.5–55.8)	50.0 (1.7–82.6)	84.6 (53.7–97.3)
NPV (95 % CI)	97.8 (96.6–98.6)	97.8 (96.6–98.6)	97.8 (96.6–98.6)	97.7 (96.5–98.6)	97.9 (96.8–98.7)	98.7 (97.7–99.3)
LR positive (95 % CI)	11.9 (3.5–40.3)	11.9 (3.5–40.3)	14.9 (4.2–52.4)	9.9 (2.2–44.1)	39.6 (10.6–148.7)	217.8 (51.2–927.5)
LR negative (95 % CI)	0.88 (0.75–1.03)	0.88 (0.75–0.84)	0.88 (0.75–1.03)	0.92 (0.81–1.04)	0.83 (0.69–1.00)	0.52 (0.35–0.77)
DOR (95 % CI)	13.5 (2.7–59.5)	13.5 (2.7–59.5)	16.9 (3.2–78.2)	10.8 (1.5–60.1)	47.7 (9.1–251.4)	416.6 (74.1–3087.2)

*TP* true positive, *FP* false positive, *FN* false negative, *TN* true negative, *CI* confidence interval, *PPV* positive predictive value, *NPV* negative predictive value, *LR* likelihood ratio, *DOR* diagnostic odds ratio

### Expert microscopy as reference test

Similar results were found when RDTs and field microscopy were compared against expert microscopy to detect any malaria (Table 2). Overall, the sensitivity remained modest for all four RDTs and for field microscopy with SD Bioline^®^ scoring better LR-positive and DOR values. When the ability to detect *P. falciparum* by HRP-2 band was assessed, First Response^®^ had a lower false positive score reflected by higher PPV and LR-positive relative to the other RDTs (Table 3). SD Bioline^®^ indices to detect *P. vivax* by pLDH band were relatively better than the other RDTs.

### RDT detection of malaria at different parasite densities

**Fig. 1:**
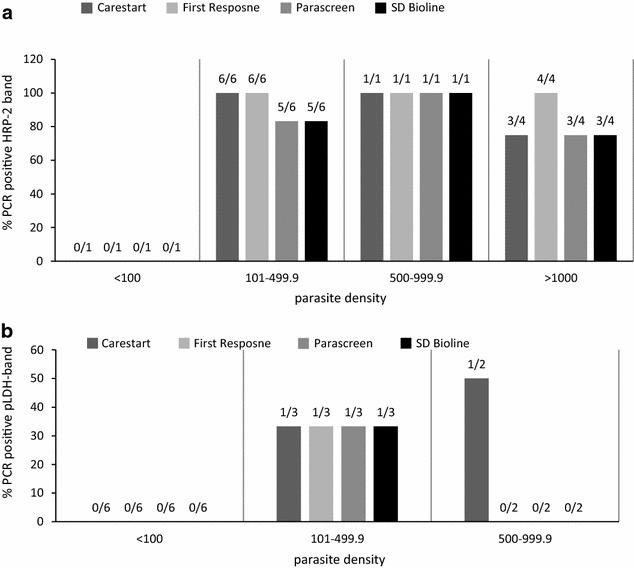
Detection of *Plasmodium falciparum* and *Plasmodium vivax* by the HRP-2 and pLDH bands at different parasite densities. **a** *P. falciparum* detection by HRP-2bands in PCR positive cases stratified by microscopy density. Figures above the *bars* are HRP-2 positive bandscorresponding to each parasite threshold out of the total 12 PCR versus expert microscopy confirmed *P. falciparum* results; excludes mixed RDT infection. **b**. *P. vivax* detection by pLDH band in PCR positive cases stratified by microscopy density. Figures above the *bars* are the pLDH bands corresponding to each parasite threshold out of the total 11 PCR versus expert microscopy confirmed *P. vivax* results; none of the PCR confirmed vivax positives had parasite density >1000/μL; excludes mixed RDT infection

The geometric mean parasite density of *P. falciparum* assessed by expert microscopy was 418 parasites/µL (range 27–13,387 parasites/uL) and of *P. vivax* was 147 parasites/µL (range 27–5733 parasites/µL). Out of the 12 PCR confirmed expert microscopy positive *P. falciparum* cases, there was one case of mono-infection with *P. falciparum* at density <100 parasite/µL and this was negative by all four RDTs. Similarly, there were 6 cases out of the 11 *P*. *vivax* PCR confirmed positives which has densities of <100 parasites/µL detected by expert microscopy; none were detected by the RDTs (Fig. 1). The ability to detect low-density *P. falciparum* infections (101–499/μL) was comparable between Carestart™ (6/6) and First Response^®^ (6/6) relative to Parascreen^®^ and SD Bioline^®^. The detection of low-density *P. vivax* infections (100–499/μL) was similar in all RDTs (33.3 %).

### End user experience

The user practicality of the RDTs was evaluated among all the 50 study midwives/nurses performing RDTs in the study. Most respondents cited the pipette of SD Bioline^®^ was the easiest to draw blood with (86.0 %) (Table 7), but to add blood into a sample well and for the overall ease of use of pipette, First Response^®^ scored 88.0 and 96.0 % respectively followed by SD Bioline^®^. The least easy pipette to draw and dispense blood with was that of Parascreen^®^. All respondents found the buffer bottles comparatively easy to use and add the right amount of drops. First Response^®^ scored highest for overall ease of use, whereas SD Bioline^®^ scored highest for clarity and readability of printed information provided with the RDT.

**Table 7:** Response frequencies of the parameters assessed for end user practicality of the four RDTs

Parameter	Carestart™	First Response^®^	Parascreen^®^	SD Bioline^®^
	n = 50	n = 50	n = 50	n = 50
Pipette use				
Easy to draw right amount of blood, n (%)	39 (78.0)	40 (80.0)	30 (60.0)	43 (86.0)
Easy to dispense blood into well, n (%)	44 (88.0)	44 (88.0)	22 (44.0)	43 (86.0)
Overall ease of use, n (%)	45 (90.0)	48 (96.0)	27 (54.0)	46 (92.0)
Buffer bottle				
Easy to dispense drops into well, n (%)	36 (72.0)	37 (74.0)	38 (76.0)	36 (72.0)
Package exterior information				
Easy to read and print clarity, n (%)	44 (88.0)	38 (76.0)	46 (92.0)	45 (90.0)
Overall ease of use^a^				
Easy, n (%)	36 (72.0)	40 (80.0)	37 (74.0)	41 (82.0)
Moderately easy, n (%)	7 (14.0)	3 (6.0)	6 (12.0)	6 (12.0)
Hard to use, n (%)	6 (12.0)	6 (12.0)	6 (12.0)	2 (4.0)

^a^One response is missing for this parameter

## Discussion

The performance of SD Bioline^®^, Carestart™, First Response^®^ and Parascreen^®^ RDTs to screen mostly asymptomatic antenatal women for malaria were evaluated in four sub-districts of South west Sumba, Indonesia as a potential alternative to field microscopy. Overall the diagnostic accuracy, suggested that First Response^®^ RDT had the best combined ability to detect both *P. falciparum* and *P. vivax* infections when compared against PCR. The diagnostic odds ratio (DOR) value for First Response^®^, as a single summary indicator of test performance, was lower than that observed for expert microscopy but higher than the 23–29 DOR values observed for the other RDTs. First Response^®^ was also considered the easiest to use by the clinic staff. Nevertheless, the overall differences in performance of the four RDTs to detect malaria were small and importantly, comparable to field microscopy. Any of the four brands would probably be suitable as a potential alternative to field microscopy or for use in the clinics where microscopy is not available.

However, of note was the low sensitivity (26–32 %) of all four RDTs (and field microscopy) relative to PCR, which detected 2–3 additional infections for each infection detected by RDT. These findings are consistent with the known limitations of this current generation of RDTs and highlight the challenge of applying them as a screening test in asymptomatic pregnant women who on average will have much lower parasite densities when infected than symptomatic malaria patients, the intended population for whom most RDTs are used. The geometric mean parasite densities in those with detectable parasites by expert microscopy were low for both species, especially for *P. vivax* (418 parasites/µL for *P. falciparum* and 147 parasites/µL for *P. vivax* by expert microscopy). None of the infections (any species) below 100 parasites/µL were detected by RDTs, around the threshold level of detection for many RDTs [25].

The sensitivity was also low when compared against expert microscopy (43.0–47.0 %, any species) and lower than reported in previous studies in asymptomatic pregnant women during pregnancy and at delivery [13]. In addition to the low mean parasite densities mentioned above, other methodological differences could explain the lower sensitivity. First, the expert microscopist declared a slide negative after examining 200 instead of the commonly used 100 high power fields, thus doubling the volume of blood that was examined to enhance the sensitivity of microscopy. Second, several previous studies showed that diagnostic studies with imperfect gold standard such as microscopy can lead to bias, the direction of which depends on the correlation between the index and reference test [30]. To overcome this, some studies comparing RDT against microscopy use PCR as the resolver test. However, when PCR is conducted on discrepant samples only, it has potential for upward bias overestimating sensitivity and specificity [31–33] compared to studies that also test a random sample of concordant negative samples as recommended by WHO [34], or conduct PCR on all samples regardless of the RDT and microscopy results, as done in this study. E.g. if only PCR was used for the discordant RDT-microscopy samples in our study, the sensitivity of the RDTs against expert microscopy would increase from 40–50 % to 75.0–87.0 %, comparable to findings in other low transmission areas such as in India where *P. falciparum* and *P. vivax* co-exist [10].

The sensitivity of the Pan-pLDH band to detect (PCR confirmed) *P. vivax* mono-infections (Table 6) was particularly low relative to PCR whereas field microscopy performed slightly better as indicated by higher DOR value (40 for field microscopy versus 10–12 for the Pan-pLDH RDTs bands). Six out of 11 infections detected by expert microscopy had densities below 100 parasites per/μL and none of them were detected by RDTs. The low parasite densities of *P. vivax* might explain the low sensitivity of the pLDH bands to detect *P. vivax*. Studies in Myanmar and Madagascar with comparable transmission level to Indonesia also found decreased accuracy of Pan-pLDH to *P. vivax* with decreasing parasite density [21, 35].

Interestingly, Pan-pLDH detected *P. falciparum* cases that were negative by the HRP-2 band resulting in a better test performance when results of both bands were combined (Table 5). It is not known whether genetic variation in *P. falciparum* histidine rich protein, such as described in parasites from the Peruvian Amazon [36], some of which lack PfHRP2 or PfHRP3, and the extensive variation observed recently as a possible cause of variable sensitivity of the HRP2 band among Indian *P. falciparum* parasite populations [37] exist in Indonesia and if they affect PfHRP concentrations.

The low parasite densities for *P. vivax* may also explain the lack of agreement between expert and field microscopy as none of the 8 smears classified as *P. vivax* by field microscopy (4 of which were PCR confirmed) were confirmed by expert microscopy, and none of the 13 *P. vivax* infections detected by expert microscopy (11 of which were PCR confirmed) were detected by field microscopy.

Comparison between other RDT studies in pregnancy and this study was problematic because of the differences in transmission level, endemic species, RDT brands and analysis method used. Earlier studies have used RDTs to diagnose placental malaria at delivery [15, 16, 38]. Most African studies have used HRP-2 based RDTs to diagnose malaria in pregnancy and assessed for *P. falciparum* detection [39–41]. Other studies that used RDTs to screen pregnant women have used different brands at different transmission settings [18, 20].

To qualify for an effective screening test used in low malaria prevalent population, the RDTs should have an acceptable balance to “rule-out” and “rule-in” infections in asymptomatic women. The RDTs demonstrated these qualities for any malaria detection and their performance overall (any species) was similar to field microscopy. The high specificity and NPV indicated that the RDTs correctly identified non-infected women, which would avoid treating women without malaria.

The perceived ease of use by the field staff for the four RDTs were comparable. When the package readability was accounted for, SD Bioline^®^ was favoured whereas First Response^®^ was preferred for overall practicality including ease of blood transfer pipette and buffer usage. The loop pipette for blood transferring found in Parascreen^®^ kit was preferred least, similar to studies elsewhere [42]. A very useful advantage of the RDTs over microscopy was that women could be shown the RDT results, which made the RDTs more acceptable to asymptomatic women and which is likely to enhance compliance with any 3-day ACT regimen required to treat the malaria infection.

Limitations of our study included the cross sectional study design, which limited determining when sub-microscopic infections may become patent and we were unable to follow women until delivery to correlate antenatal RDT findings with placental malaria. Batch quality Lot testing prior to using the RDTs was not performed. The study environment with dedicated research staff who were trained for the study and attention given to operational conditions, might not reflect the routine field situations.

## Conclusions

The diagnostic accuracy to detect malaria among mostly asymptomatic pregnant women and perceived ease of use by field staff was slightly better with First Response^®^, but overall differences between the four RDTs were small and performance was comparable to field microscopy. Best test performance to detect *P. falciparum* was achieved by combining the results of both pLDH and the HRP-2 bands. Combination RDTs are a suitable alternative to field microscopy to screen for malaria in pregnancy in rural Indonesia. However both RDTs and field microscopy missed many infections detected by PCR, especially *P. vivax* infections. The clinical relevance of these low density infection needs to be determined further, as it may help determine whether preventive strategies such as intermittent preventive therapy in pregnancy (IPTp) may be more effective than single or repeated screening with RDTs during pregnancy.
